# Telomere length and h*TERT* genetic variants as potential prognostic markers in multiple myeloma

**DOI:** 10.1038/s41598-023-43141-7

**Published:** 2023-09-22

**Authors:** Marta Dratwa, Piotr Łacina, Aleksandra Butrym, Diana Porzuczek, Grzegorz Mazur, Katarzyna Bogunia-Kubik

**Affiliations:** 1 Laboratory of Clinical Immunogenetics and Pharmacogenetics, Hirszfeld Institute of Immunology and Experimental Therapy, Polish Academy of Sciences, Wrocław, Poland; 2 Department of Cancer Prevention and Therapy, Wroclaw Medical University, Wrocław, Poland; 3 Department of Internal, Occupational Diseases, Hypertension and Clinical Oncology, Wroclaw Medical University, Wrocław, Poland

**Keywords:** Genetics, Cancer genetics, Clinical genetics, Epigenetics, Genetic markers, Genotype, Immunogenetics, Medical genetics, Cancer, Cancer genetics, Haematological cancer, Tumour biomarkers

## Abstract

Telomere dysfunction is a notable event observed in many cancers contributing to their genomic instability. A major factor controlling telomere stability is the human telomerase reverse transcriptase catalytic subunit (hTERT). Telomere shortening has been observed in multiple myeloma (MM), a plasma cell malignancy with a complex and heterogeneous genetic background. In the present study, we aimed to analyse telomere length and h*TERT* genetic variants as potential markers of risk and survival in 251 MM patients. We found that telomere length was significantly shorter in MM patients than in healthy individuals, and patients with more advanced disease (stage III according to the International Staging System) had shorter telomeres than patients with less advanced disease. MM patients with h*TERT* allele rs2736100 *T* were characterized with significantly shorter progression-free survival (PFS). Moreover, allele rs2736100 *T* was also found to be less common in patients with disease progression in response to treatment. h*TERT* rs2853690 *T* was associated with higher haemoglobin blood levels and lower C-reactive protein. In conclusion, our results suggest that telomere length and h*TERT* genetic variability may affect MM development and can be potential prognostic markers in this disease.

## Introduction

Multiple myeloma (MM) is an incurable haematologic malignancy, characterized by uncontrolled proliferation and accumulation of aberrant monoclonal plasma cells in the bone marrow^1^. The risk of developing MM increases with age, and it is more commonly diagnosed in males. The median age at diagnosis is 65 years, and the current mean survival of MM patients is approximately 5 years^2^. The symptomatic stage of MM is associated with lytic bone lesions, severe anaemia, hypercalcaemia, infections and kidney failure^1^. MM has a complex and heterogeneous genomic landscape. Its main feature is the presence of numerous genetic changes e.g. mutations, structural rearrangements and copy number variations^3^. Genomic alterations can be detected ranging from pre-malignant stages of monoclonal gammopathy of undetermined significance (MGUS) and smouldering MM (SMM) to clinical overt MM^4^.

Telomere dysfunction is one of the mechanisms that may lead to the genetic and clinical heterogeneity observed in MM. Telomeres are fragments of DNA located at the ends of linear chromosomes consisting of hexameric repeats of (5′-TTAGGG-3′)n. Chromosomes lacking telomeres may assemble abnormally and uncontrollably, causing genomic instability and changes in the karyotype^5^. In the group of MM patients, in addition to the shortening of telomere length with each cycle of cell proliferation, another important source of chromosomal instability was observed—telomere uncapping. This mechanism affects telomere structure and has been shown to be related to aggressiveness in MM at diagnosis^6,7^. Telomere length is tightly regulated by a reverse transcriptase called telomerase^5^. The primary task of telomerase is to extend the 3′ ends of chromosomes by adding short DNA fragments—telomeric repeats. Telomerase is a holoenzyme with reverse transcriptase properties, an integral component of which is the RNA template (TERC), and the human telomerase reverse transcriptase catalytic subunit (hTERT)^8^. Telomerase activity is suppressed in most human somatic cells and only retained in germ cells, activated T and B lymphocytes, and to some extent in stem cells^9,10^. Interestingly, telomerase activity is found in 90% of patients with newly diagnosed and relapsed MM. Short telomeres are observed in MM patients both at diagnosis and during disease progression. A study by Wu et al. showed that MM patients can be characterized by short telomere length and, at the same time, high levels of telomerase activity. The above observations support the concept of protection of critically short telomeric DNA by telomerase^11^. Nevertheless, it seems that the telomere shortening in haematological malignancies might still be associated with occurrence of aberrant karyotype^12,13^. In addition, inhibition of telomerase activity by GRN163L (Imetelstat), a lipid-conjugated thio-phosphoramidate oligonucleotide, was shown to be effective in the treatment of MM patients in both in vitro and in vivo studies^14^. Short telomeres may be responsible for chemotherapy resistance, as telomere shortening can result in adaptation of malignant cells, allowing their increased proliferation to overcome the cytotoxicity of treatment^15^.

The occurrence of telomere shortening also depends on the expression level, promoter mutations and genetic variability within the gene coding for h*TERT*^16,17^. The h*TERT* gene is located on the shorter arm of chromosome 5 (5p15.33) and consists of 15 introns, 16 exons and a 260 bp promoter core^18^. Aberrant gene variants may play a key role in transforming somatic cells into malignant cells by activating telomerase and other related signalling pathways, e.g. Wnt/β-catenin pathway^19,20^.

In the present study, we aimed to assess telomere length and h*TERT* genetic variants as potential markers associated with risk, survival, response to treatment and clinical course of the disease in MM. For this purpose, telomere length as well as six SNPs (rs2853690, rs2736100, rs33954691, rs35033501, rs2735940, rs10069690) located within the h*TERT* gene were analysed.

## Results

### Telomere length in multiple myeloma patients and healthy individuals

**Figure 1:**
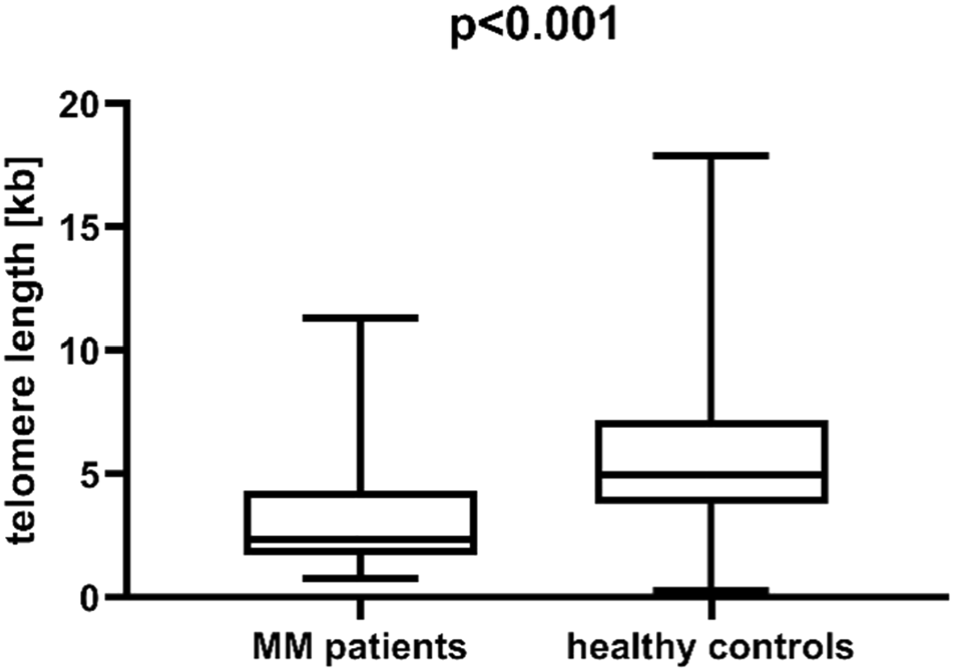
Telomere length in multiple myeloma (MM) patients and healthy individuals.

We observed that telomere length was significantly shorter in MM patients than in healthy individuals (median length 2.35 vs 4.96, p < 0.001, Fig. 1). This association was further confirmed in a multivariate generalized linear model analysis which included, alongside telomere length, also age and sex of patients and controls. Telomere length was proved to be an independent factor of MM risk (p = 0.010, Table 1).

**Table 1:** Results of a multivariate logistic regression analysis of multiple myeloma (MM) risk.

	Odds ratio (95% CI)	p-value
Telomere length	0.733 (0.579–0.928)	**0.010**
Age	1.272 (0.193–1.356)	** < 0.001**
Sex	2.069 (0.683–6.264)	0.198

Significant values are in bold.

**Figure 2:**
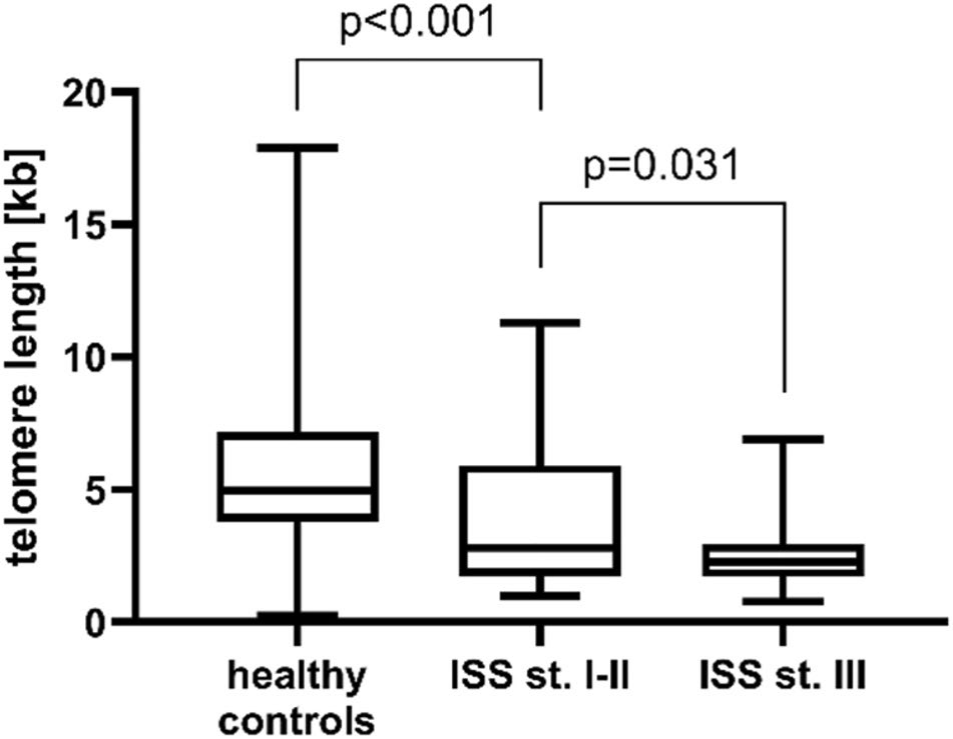
Telomere length in MM patients with less advanced (stages I–II according to the ISS) and more advanced (stage III) disease. Healthy individuals are also included for comparison. Patients with more advanced disease are characterized by shorter telomeres than those with less advanced disease, although both groups of patients have significantly shorter telomeres than healthy individuals.

Telomere length was also shorter in patients with more advanced disease (stage III according to the International Staging System; ISS) than in patients with less advanced disease (median length 2.27 vs 2.81, p = 0.031, Fig. 2). Nevertheless, patients with less advanced disease (ISS I-II) still had significantly shorter telomeres than healthy individuals (median length 2.81 vs 4.96, p < 0.001, Fig. 2). Telomere length also weakly correlated with blood albumin concentration (R = 0.293, p = 0.004). No associations with overall or progression-free survival or other clinical parameters was observed.

### h*TERT* genetic polymorphism and MM survival

**Figure 3:**
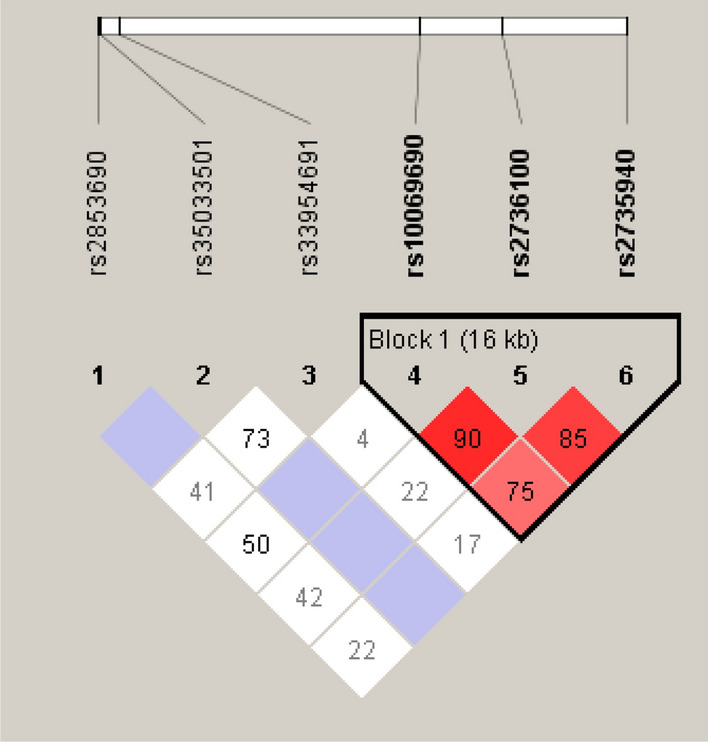
Linkage disequilibrium between h*TERT* SNPs included in the study. Darker colours represent higher D′ values. Results were obtained from the Haploview 4.2 software^21^.

Genotype frequencies for all the six analysed h*TERT* SNPs (rs2853690, rs2736100, rs33954691, rs35033501, rs2735940, rs10069690) are presented in Table 2. All the SNPs were in Hardy–Weinberg equilibrium in both patients and healthy controls. Linkage disequilibrium (LD) analysis showed that rs2736100, rs2735940, rs10069690 were in relatively high LD (Fig. 3). No difference was observed in the allele and genotype frequencies between MM patients and healthy individuals.

**Table 2:** h*TERT* genotype frequencies in multiple myeloma (MM) patients and healthy individuals.

	MM patients (N = 251)	Healthy individuals (N = 226)
rs2853690		
CC	185 (73.7%)	140 (71.8%)
CT	62 (24.7%)	55 (28.2%)
TT	4 (1.6%)	0 (0.0%)
rs2736100		
GG	58 (23.1%)	47 (20.8%)
GT	118 (47.0%)	115 (50.9%)
TT	75 (29.9%)	64 (28.3%)
rs33954691		
CC	218 (86.9%)	163 (84.0%)
CT	32 (12.7%)	28 (14.4%)
TT	1 (0.4%)	3 (1.6%)
rs35033501		
GG	240 (95.6%)	188 (96.4%)
GA	11 (4.4%)	7 (3.6%)
AA	0 (0.0%)	0 (0.0%)
rs2735940		
TT	61 (24.3%)	48 (21.3%)
TC	125 (49.8%)	111 (49.3%)
CC	65 (25.9%)	66 (29.3%)
rs10069690		
GG	141 (56.2%)	123 (54.4%)
GA	93 (37.0%)	89 (39.4%)
AA	17 (6.8%)	14 (6.2%)

**Figure 4:**
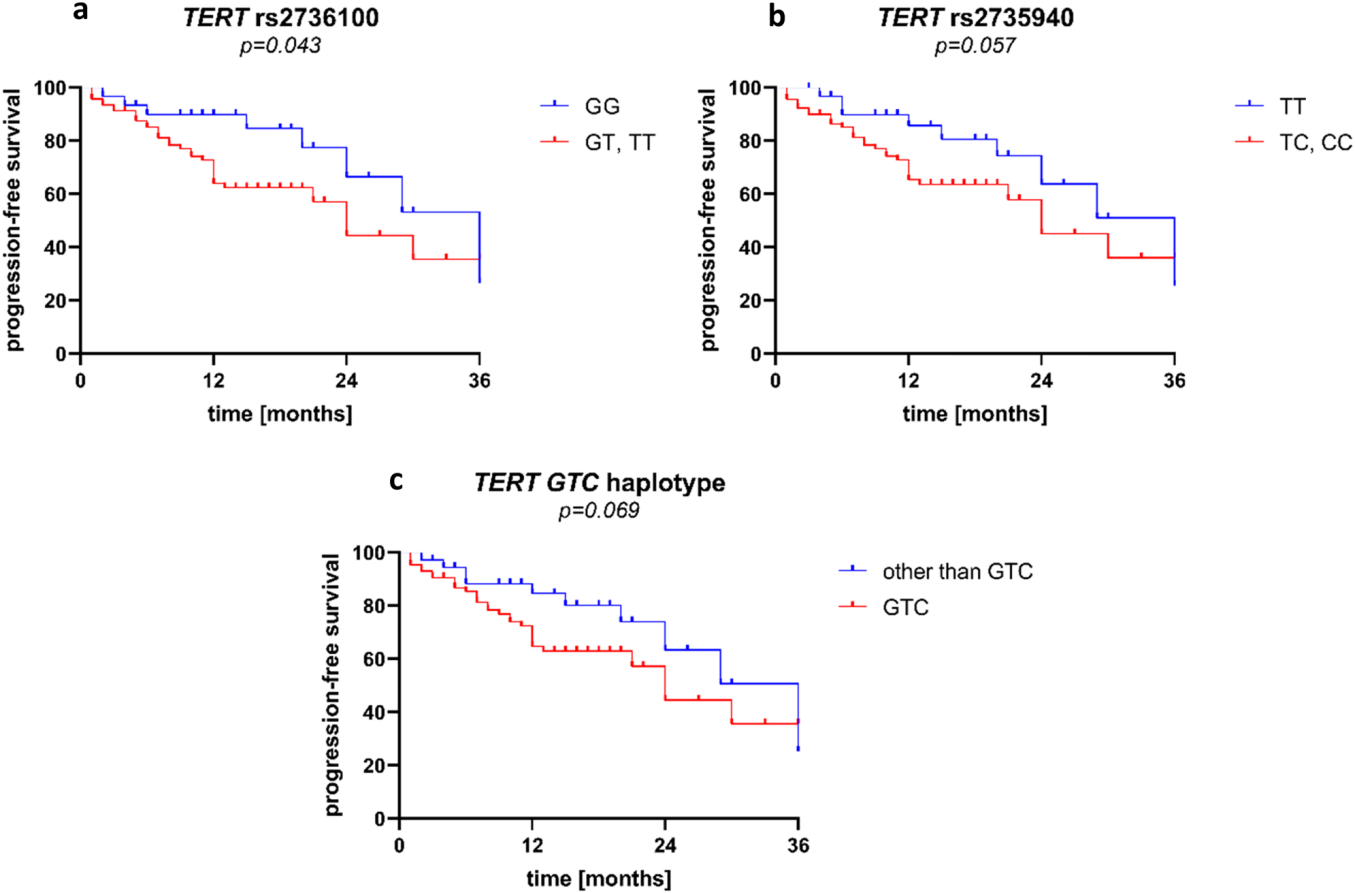
Progression-free survival during the first 3 years in multiple myeloma patients and h*TERT* SNPs rs2736100 (**a**), rs2735940 (**b**), as well as the *GTC* (rs10069690 *G*, rs2736100 *T*, rs2735940 *C*) haplotype (**c**).

We performed an analysis of both overall (OS) and progression-free survival (PFS) to test if any of the h*TERT* SNPs might affect it. No association with OS was observed. We noticed that alleles rs2736100 *T* and rs2735940 *C* corresponded with shorter PFS during the first 3 years, but longer PFS thereafter (Supplementary Fig. S1). When analysing only early survival (first 3 years), patients with allele rs2736100 *T* were characterized with significantly shorter PFS than patients without this allele (p = 0.043, Fig. 4a). A similar association, although not statistically significant, was also observed for allele rs2735940 *C* (p = 0.057, Fig. 4b) and for the *GTC* (rs10069690 *G*, rs2736100 *T*, rs2735940 *C*, based on the LD data as shown in Fig. 3) haplotype (p = 0.069, Fig. 4c). These observations were confirmed in analyses in a Cox proportional hazards regression model including age and later ISS stages in addition to each of the risk alleles/haplotypes. Allele rs2736100 *T* (p = 0.035), but not allele rs2735940 *C* (p = 0.102) or haplotype *GTC* (p = 0.075), was found to be an independent factor of shorter PFS.

**Figure 5:**
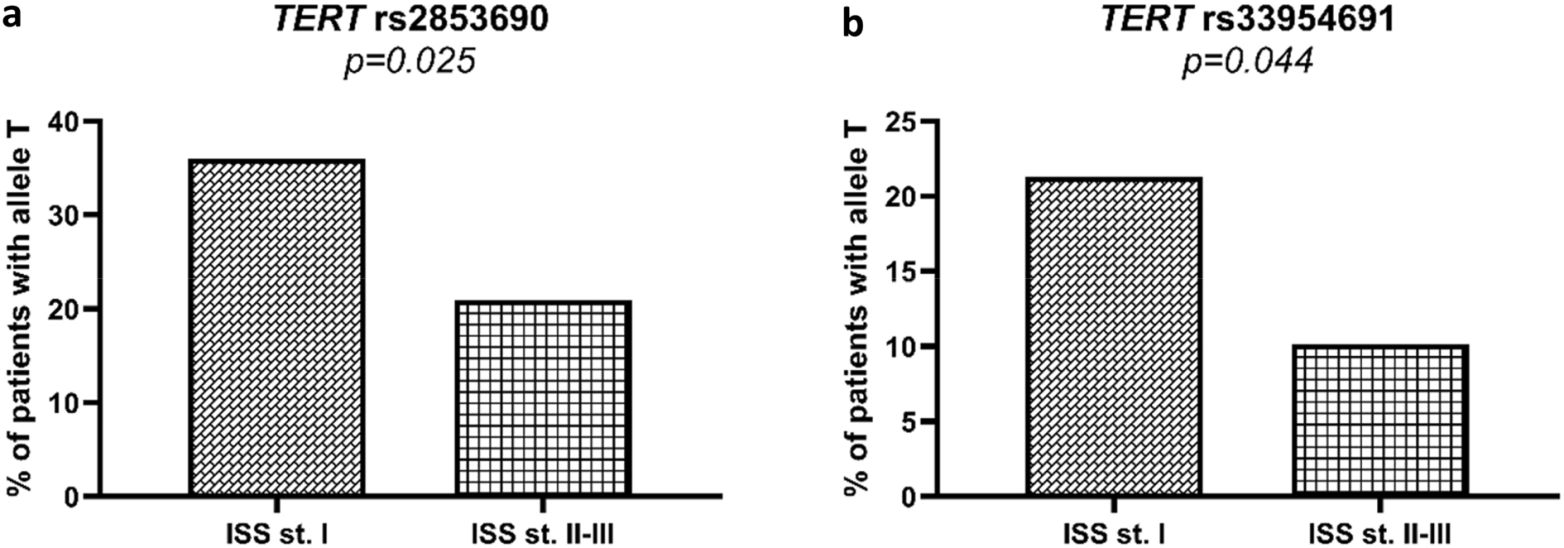
Frequency of alleles rs2853690 *T* (**a**) and rs33954691 *T* (**b**) among patients with more and less advanced disease at diagnosis according to the ISS criteria. Both alleles are more common in patients with ISS I.

When stratified according to the ISS criteria, patients with less advanced disease (ISS I) on diagnosis more commonly carried the rs2853690 *T* allele than those diagnosed with more advanced (ISS II-III) disease (22/61 vs 35/167, p = 0.025, Fig. 5a). Similarly, patients with less advanced disease (ISS I) also carried allele rs33954691 *T* more commonly than other patients (13/61 vs 17/167, p = 0.044, Fig. 5b). Detailed information on distribution of *hTERT* genotypes in patients with different ISS stages is shown in Supplementary Table S1. Allele rs2853690 *T* was also associated with higher haemoglobin blood levels (median value 11.7 vs 10.7 g/dL; p = 0.006) and lower C-reactive protein (1.8 vs 4.6 mg/dL; p = 0.034) compared to patients without this allele. Interestingly, we also observed that alleles rs2736100 *T*, rs2735940 *C*, and haplotype *GTC* were less common in patients with progressive disease in response to treatment (p = 0.024, p = 0.020, p = 0.031 for the two alleles and the *GTC* haplotype, respectively).

We also analysed differences in telomere length between carriers of various *hTERT* alleles. MM patients with alleles rs10069690 *G* had longer telomeres than patients without this allele (2.33 vs 3.99 kb, p = 0.047). No associations with other SNPs included in this study were observed.

## Discussion

The current knowledge of human cancer development shows that telomere dysfunction may be a key event causing genomic instability and disease progression in many types of solid tumours e.g. renal cell carcinoma^22^, glioma^23,24^, esophageal squamous cell carcinoma^25^, gastric cancer^26^, ovarian and breast cancer^27–30^. Additionally, telomere shortening has also been observed in haematological malignancies, both acute and chronic leukaemias^31–34^, some lymphomas^35–37^, as well as bone marrow failure syndromes (myelodysplastic syndrome (MDS))^38,39^ and aplastic anaemia^40–42^. It is not surprising that diseases related to telomere length manifest so prominently in the bone marrow, given that it is a place where turnover of cells is remarkably high, with app. 10^9^ cells being produced every hour^43^. Haematopoiesis is based on the ability of stem cells to self-renewal and differentiation. In humans, there is clear evidence that short telomeres cause quantitative and qualitative defects in HSCs that manifest as stem cell depletion^44–47^.

MM is a very complex haematologic malignancy with a heterogenous genomic landscape, involving accumulation of mutations, as well as structural and copy number changes. These genetic variations occur in combination with point mutations and affect various cellular pathways, including genome maintenance^3^. Telomere dysfunction is one of the mechanisms that may lead to the genetic and clinical heterogeneity observed in MM, therefore analysis of telomere length may have prognostic significance. Our present study was conducted on a group of newly diagnosed untreated MM patients. We observed that significant shortening of telomere length in MM patients compared to healthy individuals. This is contrast to a study by Campa et al., as they found a link between longer telomeres and an increased risk of MM^48^. However, most other studies seem to confirm our observation. Similar results were reported by Wu et al. in a study analysing CD138 positive cells isolated from the bone marrow of patients with newly diagnosed or relapsed MM and healthy donors. These results showed significantly reduced telomere length in MM patients compared to telomere length in plasma cells from healthy donors^11^. Cottliar et al. studied bone marrow cells from patients with MM and with MGUS. Similarly to our results, they observed short telomeres in MM patients at diagnosis and during relapse. Additionally, they noted that telomere length in bone marrow cells was restored after disease remission^49^. In a three-dimensional (3D) telomere analysis, Klewes et al. showed changes in 3D nuclear architecture during disease progression from MGUS to MM, resulting in increased telomere attrition and consequent shorter telomeres in MM, as well as in relapsed MM, compared to MGUS^50^.

Hyatt et al. stratified their group of MM patients using a length threshold for telomere dysfunction (telomere length below 3.81 kb) previously defined by Lin et al.^51,52^. They showed that MM patients with short telomeres (< 3.81 kb) had significantly shorter overall survival. In their study, short telomeres were also associated with significantly worse survival in high-risk ISS patients. Moreover, in a multivariate modelling analysis, telomere length as well as patient’s age and ISS were identified as predictors of MM risk. Based on these data, Hyatt et al. proposed that each ISS prognostic subset could be further stratified for risk according to telomere length, supporting the inclusion of this parameter as a refinement of the ISS system^51^.

Telomere length can be influenced by genetic variability, including mutations in the telomerase components h*TERT* and *TERC*, and various single-nucleotide polymorphisms (SNPs), some of which are located near genes with known roles in telomere maintenance^53^. In addition, SNPs can affect telomere length, as their variants can modulate the expression of a broad spectrum of genes e.g. *Myc*, *TP53* and *NFKB*^30,54,55^. For example, − 1327C>T (rs2735940) is a SNP located in the promoter region of h*TERT* gene (h*TERT*p) and is a T/C transition 1327-bp upstream of the transcription start site and is able to induce expression of h*TERT* by upregulating its transcriptional activity in vitro and h*TERT* mRNA expression in vivo^53^. This SNP has been extensively analysed in various types of solid cancers. However, it has not been studied much in haematological diseases, except for one study on patients with childhood ALL^56^. In this study, we observed that MM patients carrying* C* allele h*TERT*p rs2735940 had shorter PFS during the first 3 years. Sheng et al. reported that the *TT* genotype and *T* allele were associated with ALL in Chinese children^56^. However, conflicting results were obtained by Eskandari et al., who concluded that rs2735940 does not increase the risk of ALL^57^. In patients with renal cell carcinoma, the *CC* genotype h*TERT*p rs2735940 was associated with shorter time to disease progression and shorter overall survival^58^. In our present study, we did not observe any significant associations between rs2735940 and telomere length. However, there are reports suggesting that h*TERT* rs2735940 polymorphism affects telomere length and that longer telomeres were associated with an increased breast cancer (BC) and lung cancer risk^59,60^. Our previous study showed that women with BC and the *CC* genotype had longer telomeres than those with *TC* and *TT* genotypes. Additionally, BC patients with the rs2735940 *C* allele were characterized by more invasive tumours than patients with the *TT* genotype^30^.

The next SNP, rs2736100, is located in intron 2 of the h*TERT* gene and based on the result of the Evolutionary and Sequence Pattern Extraction through Reduced Representation score, it is located within a putative regulatory region^61,62^. It is the most frequently analysed polymorphic variant of h*TERT*. The *C* allele of rs2736100 was found to be associated with longer telomeres, which is consistent with the direct regulatory effect of the rs2736100 genotype on h*TERT* gene expression^29^. Interestingly, the effect strength of the rs2736100 polymorphism may vary between populations, as demonstrated, e.g. in Swedish and Chinese males with myeloproliferative neoplasms (MPNs)^63^. In addition, rs2736100 *C* is also associated with increased blood cell count, a hallmark of MPN^64,65^, and has been identified as a risk variant for MPN in the Icelandic population^64^. Tong et al. observed a higher frequency of the *CC* genotype and *C* allele in Chinese AML patients^66^. Furthermore, Rampazzo et al. observed longer telomeres at diagnosis and greater telomere erosion during neoadjuvant chemoradiotherapy in patients with rectal cancer with *CC* genotype compared to patients with the other genotypes^67^. In the present study, we observed that the *T* allele rs2736100 was associated with shorter early PFS in MM patients. We confirmed this observation (for* T* allele rs2736100) by a Cox proportional hazards regression model analysis, including age and later ISS stages in addition to each of the risk alleles. In our previous study, patients with CLL carrying *C* allele were characterized by longer telomeres with less advanced disease (Rai 0–I or Binet A) compared to patients with the *C* allele, but exhibiting more advanced stage of CLL^32^. In women with BC we showed that patients with *T* allele rs2736100 had more invasive tumours than BC patients with other genotypes^30^.

Another SNP, rs10069690, was much more extensively studied. It is known to act as a risk factor in many cancers^68^ and was found to be a marker of decreased risk for MM in an earlier study^48^. However, we were not able to confirm this association in our present study. Three other SNPs included in our study were predicted to affect either miRNA binding (rs2853690) or splicing (rs33954691, rs35033501) based on an analysis by the National Institute of Environmental Health Sciences SNP Function Prediction tool^69^. Of these, we found rs2853690 *T* and rs33954691 *T* to be more common in patients with less advanced disease (lower ISS stage). Additionally, rs2853690 *T* was also associated with higher haemoglobin and lower C-reactive protein levels, low haemoglobin and high CRP being common features of MM progression^70,71^. All three of those SNPs have been relatively poorly studied. The rs2853690 is known to affect circulating h*TERT* mRNA levels and response to treatment in patients with rectal cancer, as well as spontaneous preterm labour in pregnant women^67,72^. rs33954691 was shown to affect longevity and leucocyte telomere length^73,74^. It was also associated with increased risk of radioiodine-refractory papillary thyroid carcinoma^75^. All three SNPs were analysed in our previous study on chronic lymphocytic leukaemia (CLL), although only rs35033501 was associated with CLL risk^32^.

The regulation of the h*TERT* gene is a very complex process and h*TERT*p can be activated by multiple mechanisms in haematological malignancies. Moreover, genetic variation in h*TERT* may modulate telomere length and thus such genetic variants may be risk factors for cancer development. So, it appears that the optimal telomere length is a balance of cell proliferation, senescence and control. Shortening of telomeres to critical length results in a loss of telomere protection, leading to chromosomal instability, which can contribute to the abnormalities in the hematopoietic process.

## Methods

### Patients and controls

The study included 251 newly diagnosed Polish MM patients and 226 healthy blood donors serving as the control group. Both groups were nearly equally divided into men and women (the ratio of females was 0.502 and 0.420, respectively). Blood samples were collected at diagnosis after obtaining informed consent from patients. All methods were used according to the Declaration of Helsinki. The study was approved by the Wroclaw Medical University Bioethical Committee (ethical approval code: 369/2019). According to International Staging System (ISS) stratification, 61 (24.3%) patients were in stage I at diagnosis, 76 (30.3%) were in stage II, 91 (36.3%) were in stage III, and 23 lacked data on ISS. Most patients were administered either cyclophosphamide, thalidomide, dexamethasone (CTD)—38.8%, bortezomib, thalidomide, dexamethasone (VTD)—13.2%, or bortezomib, melphalan, prednisone (VMP)—11.0% as first line therapy. Response to treatment was as follows: complete response (n = 43), very good partial response (n = 32), partial response (n = 81), minor response (n = 7), stable disease (n = 20), progressive disease (n = 19).

### DNA extraction

Genomic DNA was isolated from peripheral blood taken on EDTA using Maxwell 16 Blood DNA Purification Kit (Promega Corporation, Madison, WI, USA) and the Qiagen DNA Isolation Kit (Qiagen, Hilden, Germany) following the recommendation of the manufacturers. DNA concentration and purity were quantified on a DeNovix DS-11 spectrophotometer (DeNovix Inc., Wilmington, DE, USA). The isolated DNA was then stored at − 20 °C until h*TERT* genotyping and evaluation of the telomere length in MM patients and healthy individuals.

### Genotyping of h*TERT* gene polymorphisms

The selection of the studied single nucleotide polymorphisms (SNPs) within the h*TERT* gene was based on results of the SNP Function Prediction tool available on the website of the National Institute of Environmental Health Sciences (NCBI Database), as well as other auxiliary databases (https://snpinfo.niehs.nih.gov/snpinfo/snpfunc.html (accessed on 9 January, 2023); https://www.ncbi.nlm.nih.gov/snp/ (accessed on 9 January, 2023); https://www.ensembl.org/index.html (accessed on 9 January, 2023). The following criteria were used: minor allele frequency in Caucasians above 10%, change in RNA and/or amino acid chain, potential splicing site and/or miRNA binding site. One SNP (rs35033501) was additionally included based on results from previous studies^32^.

Based on the above criteria, six h*TERT* SNPs were selected for the study: rs35033501 (G>A) located in exon 16; rs33954691 (C>T) located in exon 14; rs2853690 (C>T) located in the 3ʹ untranslated region (3′UTR); rs10069690 (G>A) located in intron 4; rs2736100 (G>T) located in intron 2; rs2735940 (T>C) located in the promoter region − 1327 bp upstream of the transcription start site. The h*TERT* polymorphisms were determined by LightSNiP typing assays (TIB MOLBIOL, Berlin, Germany) using quantitative polymerase chain reaction (qPCR). Amplifications were performed on a LightCycler480 II Real-Time PCR system (Roche Diagnostics International AG, Rotkreuz, Switzerland) according to the recommendations of the manufacturer. The PCR conditions were as follows: 95 °C for 10 min followed by 45 cycles of 95 °C for 10 s, 60 °C for 10 s and 72 °C for 15 s. PCR was followed by one cycle of 95 °C for 30 s, 40 °C for 2 min and gradual melting from 75 to 40 °C.

### Quantification of telomere length

Mean telomere length was measured in the genomic DNA samples of 112 MM patients and 185 healthy controls. The group was almost equally divided into men and women (the ratio of females was 0.455). According to ISS stratification, 29 (25.9%) patients were in stage I, 39 (34.8%) were in stage II, 43 (38.4%) were in stage III, and 1 lacked data on ISS. The DNA samples were diluted with nuclease-free water to a concentration of 5 ng/mL. Telomere length measurements were performed on a LightCycler480 II Real-Time PCR system (Roche Diagnostics International, Rotkreuz, Switzerland) using qPCR test kits (ScienCell’s Absolute Human Telomere Length Quantification qPCR Assay Kit [AHTLQ], Carlsbad, CA, USA), as previously described by Dratwa et al.^76^. The PCR conditions were as follows: 95 °C for 10 min followed by 32 cycles of 95 °C for 20 s, 52 °C for 20 s and 72 °C for 45 s. Data analysis was conducted according to the manufacturer’s instructions. All reactions were run in three replicates.

### Statistical analysis

The null hypothesis that there is no difference between the frequency of alleles and genotypes between patients and controls was verified with the Fisher’s exact test, calculated using the online tool http://vassarstats.net/tab2x2.htm (version as of 3 April, 2023). Mann–Whitney U test and logistic regression model were used to compare telomere length between patients and controls, and to check for associations between various clinical parameters and presence of various genetic variants. Correlations between telomere length and clinical parameters were assessed by Spearman’s coefficient. Survival of patients was analysed using the Gehan-Breslow-Wilcoxon test and Kaplan–Meier curves, as well as the Cox proportional hazards model. All of these analyses were conducting using the Real Statistics Resource Pack for Microsoft Excel 2013 version 15.0.5023.1000 (Microsoft, Redmond, WA, USA), RStudio (RStudio, PBC., Boston, MA, USA), and GraphPad Prism (version 8.0.1, GraphPad Software, San Diego, CA, USA). P-values < 0.05 were considered statistically significant, while those between 0.05 and 0.10 were indicative of a trend.

### Supplementary Information

Supplementary Information.

## Data Availability

The datasets generated during and/or analysed during the current study are available from the corresponding author on reasonable request.
