# Supplementary material for: Telomere length and hTERT genetic variants as potential prognostic markers in multiple myeloma
Source: Sci Rep. 2023 Sep 22;13:15792. doi: 10.1038/s41598-023-43141-7 (PMC10517131; doi:10.1038/s41598-023-43141-7)
Supplement: Supplementary file 1 — Supplementary Information. [file 41598_2023_43141_MOESM1_ESM.docx]

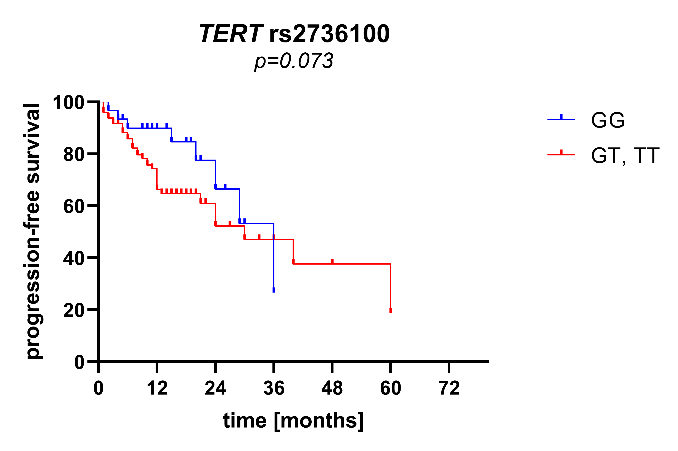

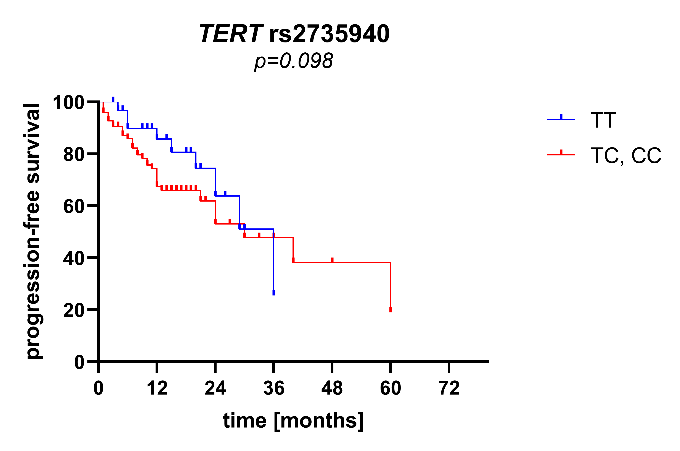

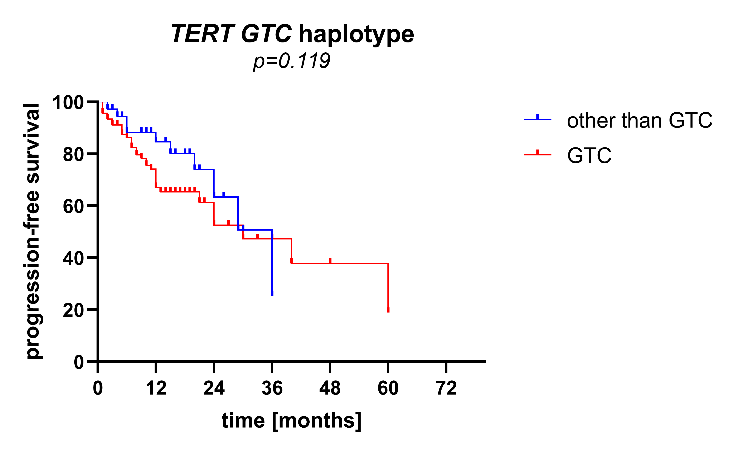


**b**

**a**

**c**

**Supplementary Figure S1**. Progression-free survival in multiple myeloma patients and h*TERT* SNPs rs2736100 (**a**), rs2735940 (**b**), as well as the *GTC* (rs10069690 *G*, rs2736100 *T*, rs2735940 *C*) haplotype (**c**).

**Supplementary Table S1.** h*TERT* genotype frequencies in multiple myeloma (MM) patients in different International Staging System (ISS) stages.

|  | **ISS-1 (N=61)** | **ISS-2 (N=76)** | **ISS-3 (N=91)** |
| --- | --- | --- | --- |
| rs2853690 |  |  |  |
| CC | 39 (63.9%) | 57 (75.0%) | 75 (82.4%) |
| CT | 20 (32.8%) | 18 (23.7%) | 15 (16.5%) |
| TT | 2 (3.3%) | 1 (1.3%) | 1 (1.1%) |
| rs2736100 |  |  |  |
| GG | 14 (23.0%) | 21 (27.6%) | 20 (22.0%) |
| GT | 29 (47.5%) | 32 (42.1%) | 42 (46.2%) |
| TT | 18 (29.5%) | 23 (30.3%) | 29 (31.9%) |
| rs33954691 |  |  |  |
| CC | 48 (78.7%) | 69 (90.8%) | 81 (89.0%) |
| CT | 12 (19.7%) | 7 (9.2%) | 10 (11.0%) |
| TT | 1 (1.6%) | 0 (0.0%) | 0 (0.0%) |
| rs35033501 |  |  |  |
| GG | 59 (96.7%) | 72 (94.7%) | 87 (95.6%) |
| GA | 2 (3.3%) | 4 (5.3%) | 4 (4.4%) |
| AA | 0 (0.0%) | 0 (0.0%) | 0 (0.0%) |
| rs2735940 |  |  |  |
| TT | 14 (23.0%) | 23 (30.3%) | 19 (20.9%) |
| TC | 31 (50.8%) | 34 (44.7%) | 46 (50.5%) |
| CC | 16 (26.2%) | 19 (25.0%) | 26 (28.6%) |
| rs10069690 |  |  |  |
| GG | 33 (54.1%) | 41 (53.9%) | 53 (58.2%) |
| GA | 24 (39.3%) | 29 (38.2%) | 32 (35.2%) |
| AA | 4 (6.6%) | 6 (7.9%) | 6 (6.6%) |
